# Interaction analysis data of simulation gaming events using the serious game Aqua Republica

**DOI:** 10.1016/j.dib.2018.06.031

**Published:** 2018-06-27

**Authors:** Steven Jean, Wietske Medema, Jan Adamowski, Chengzi Chew, Patrick Delaney, Arjen Wals

**Affiliations:** a Department of Bioresource Engineering, McGill University, Ste Anne de Bellevue, Quebec, Canada; b DHI Canada, 336 Eagle Street North, Unit 1A2, Cambridge, ON, Canada N3H 1C2; c Department of Social Sciences, Education and Competence Studies, Wageningen University, Wageningen, The Netherland

## Abstract

The data presented in this article is related to the research article entitled ‘Serious games as a catalyst for boundary crossing, collaboration and knowledge co-creation in a watershed governance context’ (Jean et al., In press) [1]. Understanding the team dynamics related to serious game simulations is critical for understanding the potential uses and functions of these simulations for knowledge co-creation (Medema et al., 2016) [2]. The data was obtained from four independent serious game simulation events and consists of *n* = 40 participants. Participants were divided into small teams and were then recorded playing the serious game Aqua Republica (http://aquarepublica.com/). Interactions were tallied and interaction maps created using the visualization software GEPHI (https://gephi.org/). The interaction maps allow for a visual representation of the progression of interactions over the course of four subsequent phases of gameplay (Jordan and Henderson, 1995) [3].

**Specifications Table**

**Table:** 

Subject area	Water resource management, social sciences, learning sciences
More specific subject area	Serious game simulations
Type of data	Table, figures
How data was acquired	Audiovisual recordings then transformed using GEPHI visualization software
Data format	Raw, analyzed and descriptive data
Experimental factors	• Sample consisted of a variety of stakeholders working in the field of watershed management • Teams were recorded playing the serious game Aqua Republica • Number of interactions of players in each team were tallied
Experimental features	Tallied interactions were used to create interaction maps
Data source location	Montreal, Canada; Ottawa, Canada; Moncton, Canada; Halifax, Canada.
Data accessibility	Data is included in this article
Related research article	Jean S, Medema W, Adamowski J, Chew C, Delaney P, and Wals A. Serious games as a catalyst for boundary crossing, collaboration and knowledge co-creation in a watershed governance context. (in press)

**Value of the data**

• Data presented in this Data in Brief transform interactions between participants into interaction maps that allow for a quick visual understanding of interaction dynamics.
• A visual understanding of the interaction data allows for trends to be spotted that may not be obvious using only numerical data.
• Data and methods included can be used for a variety of different fields and are not limited to serious game simulations or a watershed governance context.
• All studies on team and group dynamics can find value in this data and the methods used to visualize team interactions (Fig. 1, Fig. 2, Fig. 3).

**Fig. 1:**
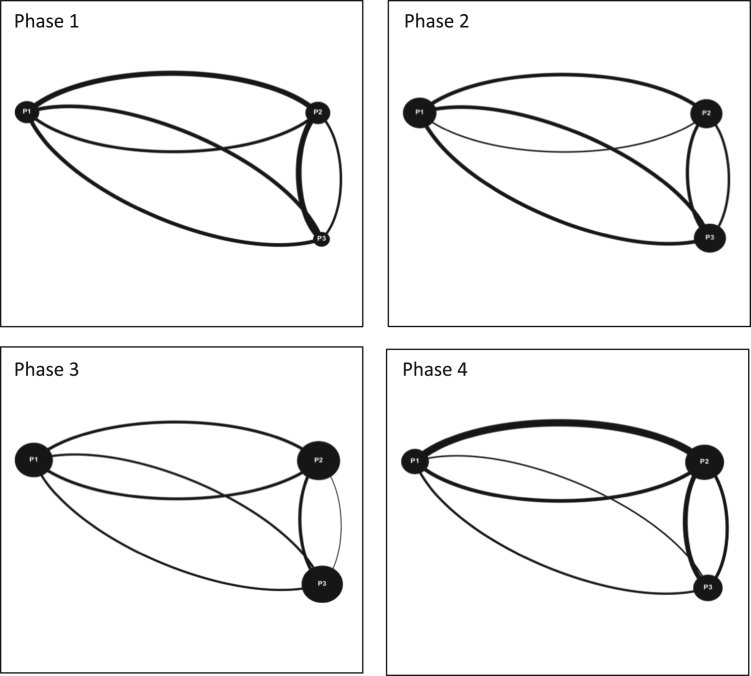
Interaction maps Moncton Team 1 Phase 1–4.

**Fig. 2:**
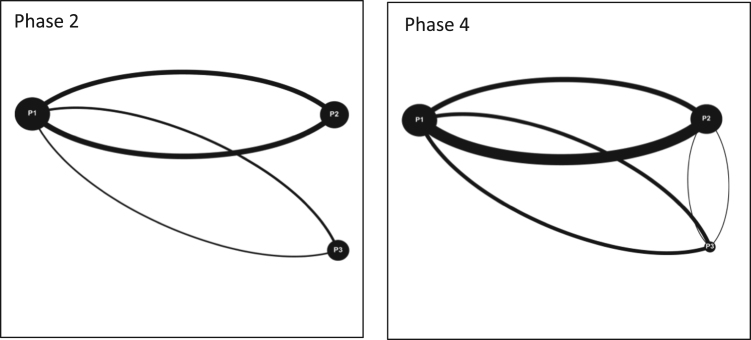
Interaction maps Moncton Team 2 Phase 2 and 4.

**Fig. 3:**
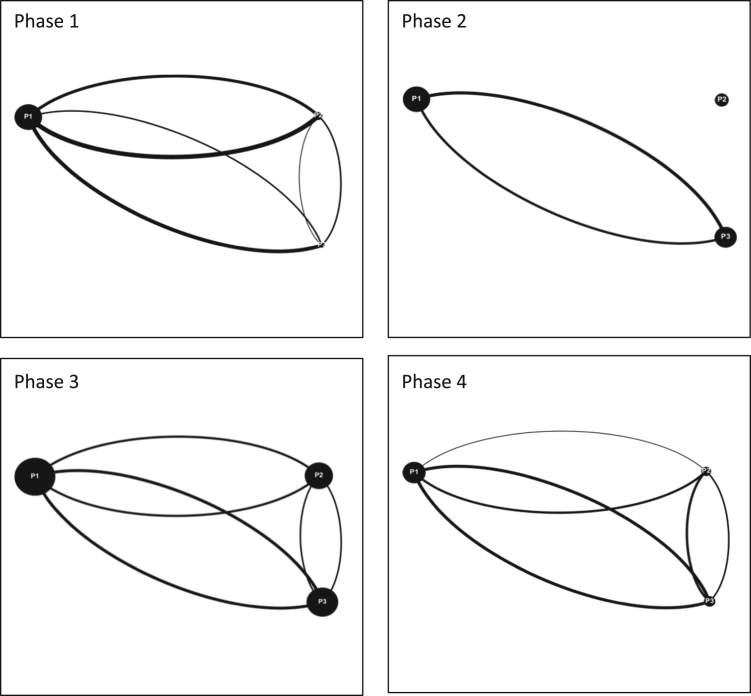
Interaction maps Halifax Team 1 Phases 1–4.

## 1 Data

**Fig. 4:**
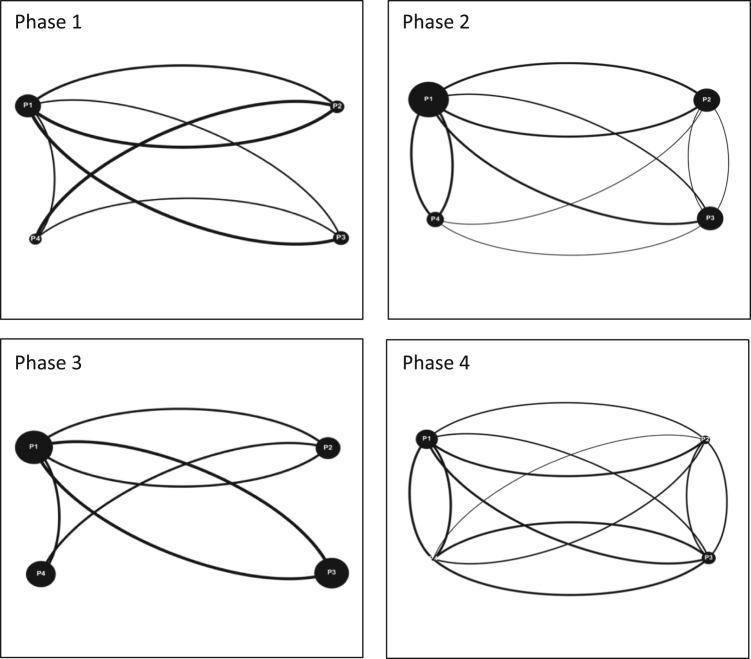
Interaction maps Halifax Team 2 Phases 1–4.

Audiovisual recordings were used in order to obtain the raw data. Interactions between players were tallied and classified as either (a) directed interactions (between two individuals) or (b) team interactions (broader statements shared with the team) [3], [4], [5]. Team interactions were documented over four phases of gameplay for each game simulation event, for each team (11 teams total). Each of the four phases of gameplay consists of a ten-minute period and is separated from the next phase by another ten-minute period. These four phases are selected over the course of each game simulation event as follows: phase 1 (0–10 min); phase 2 (20–30 min); phase 3 (40–50 min); and phase 4 (60–70 minutes). By dividing the game simulation events into these smaller phases; interactions can be tallied and displayed graphically to provide a visual overview of how team interactions evolve over time [6]. The legend for all the figures included in this Data in Brief is provided in Fig. 4 in the research article entitled ‘Serious games as a catalyst for boundary crossing, collaboration and knowledge co-creation in a watershed governance context’ [1]. Each of the following Tables and Figures (interaction maps) corresponds to one of the eleven teams. Some of the interaction maps for certain teams and specific phases of the game simulation events have been left out of this Data in Brief (i.e. Moncton Team 2 Phases 1 and 3, Ottawa Team 2 Phases 1 and 2, Ottawa Team 3 Phases 1 and 2, and McGill Team 2 Phases 1 and 3) while they have already been provided in the above mentioned research article [1].

## 2 Experimental design, materials, and methods

### 2.1 Study area and participants

**Fig. 5:**
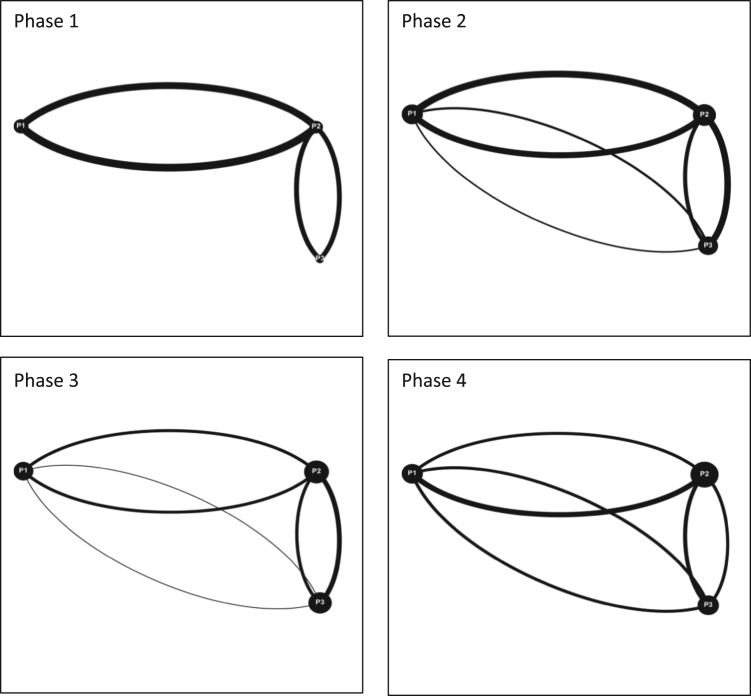
Interaction maps Ottawa Team 1 Phases 1–4.

**Fig. 6:**
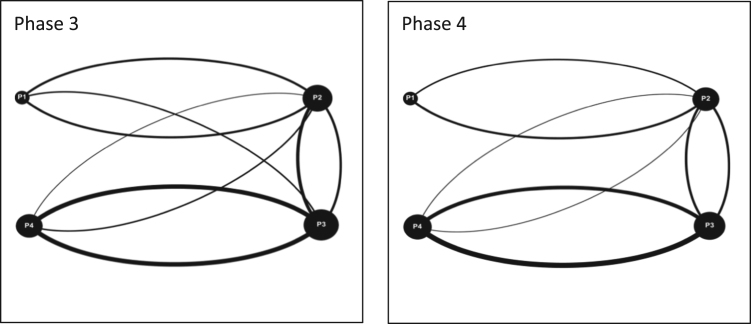
Interaction maps Ottawa Team 2 Phase 3 and 4.

**Fig. 7:**
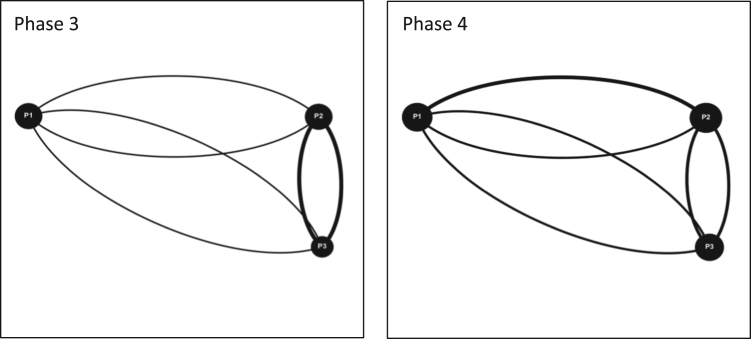
Interaction maps Ottawa Team 3 Phase 3 and 4.

**Fig. 8:**
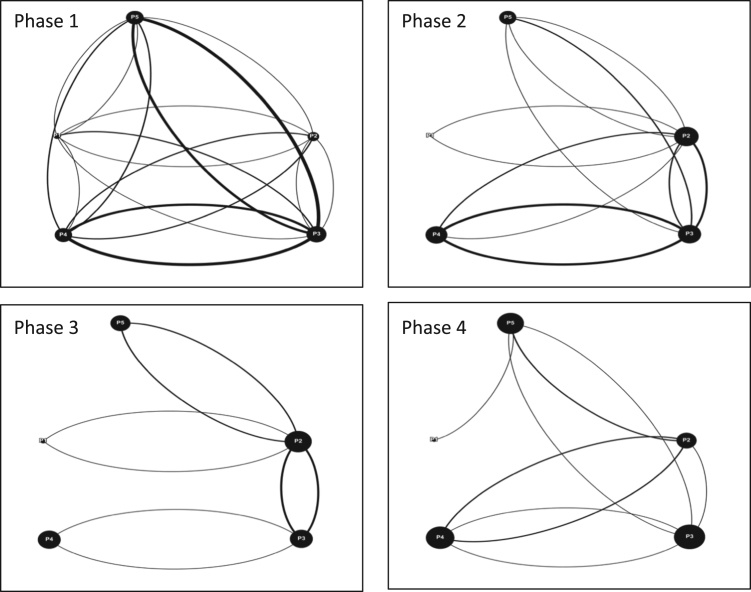
Interaction maps McGill Team 1 Phases 1–4.

**Fig. 9:**
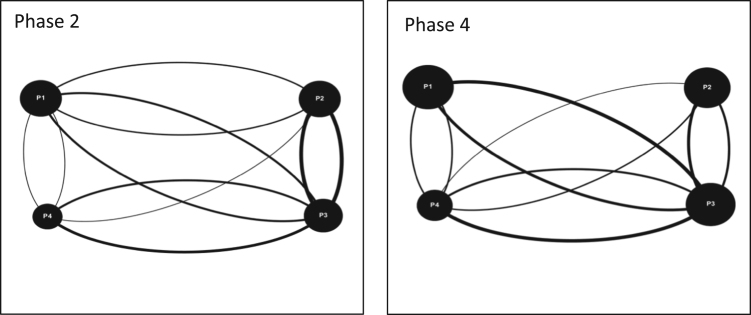
Interaction maps McGill Team 2 Phase 2 and 4.

**Fig. 10:**
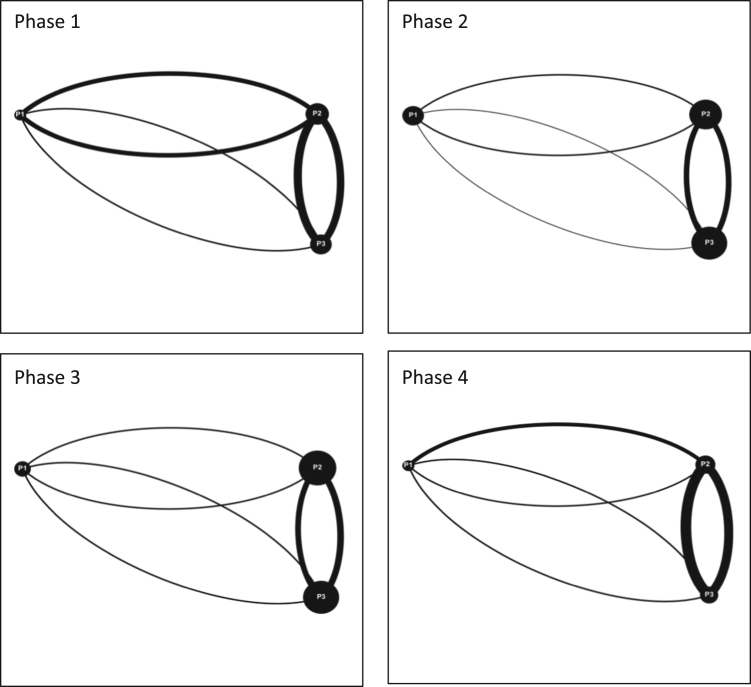
Interaction maps McGill Team 3 Phases 1–4.

**Fig. 11:**
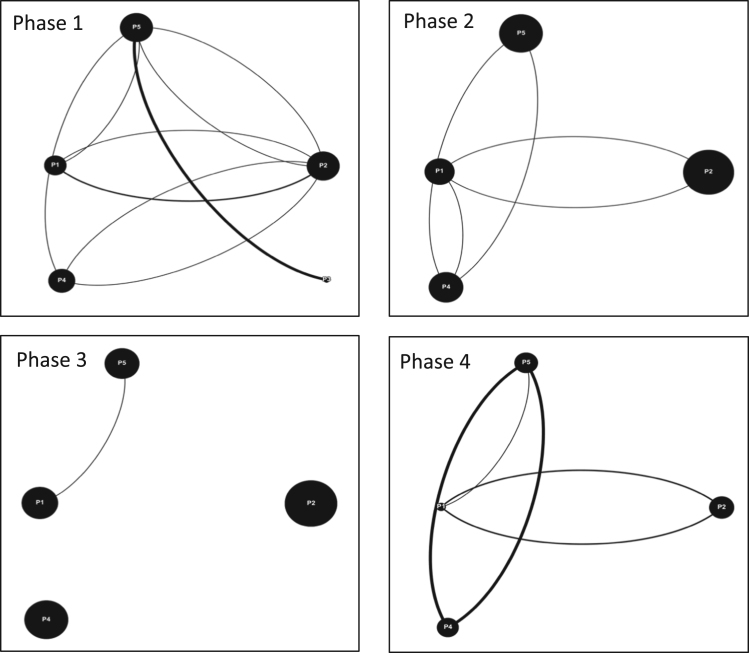
Interaction maps McGill Team 4 Phases 1–4.

As part of this data [1], four game simulation events were organized in Quebec, Ontario and the Maritimes. In Quebec, an event was organized with students from the Integrated Water Resource Management (IWRM) master׳s program at McGill University in Montreal as part of one of their required courses, this particular event was divided into two sessions with two cohorts of students. Two events took place in the Maritimes in association with two local watershed organizations acting as intermediaries for diverse stakeholder teams in their watershed territories, the Petitcodiac Watershed Alliance (PWA) in Moncton and the Sackville River Association (SRA) in Halifax. Both events involved participants from academia, local government, non-profit organizations and conservation authorities. The fourth event was organized in Ontario with the Rideau Valley Conservation Authority (RVCA) in Ottawa, involving employees, stakeholders and members of the board of directors. In total, over the course of the four events, 40 individuals participated in this data. The following table shows a breakdown of the events and the corresponding teams formed from them: (Fig. 5, Fig. 6, Fig. 7, Fig. 8, Fig. 9, Fig. 10, Fig. 11, Table 12).

### 2.2 Materials

For each event laptops were used in order to run the Aqua Republica simulations. Furthermore, camcorders were set up in front of each team in order to obtain audio-visual information for the entire time of gameplay (Table 1, Table 2, Table 3, Table 4, Table 5, Table 6, Table 7, Table 8, Table 9, Table 10, Table 11, Table 12).

**Table 1:** Interaction data Moncton Team 1.

**Moncton Team #1**									
**Phase 1**					**Phase 2**				
	P1	P2	P3	Team		P1	P2	P3	Team
P1	X	7	5	15	P1	X	5	5	21
P2	4	X	3	15	P2	2	X	3	20
P3	5	7	X	10	P3	5	4	X	20
**Phase 3**					**Phase 4**				
	P1	P2	P3	Team		P1	P2	P3	Team
P1	X	4	3	24	P1	X	10	2	17
P2	4	X	1	27	P2	5	X	4	24
P3	3	4	X	26	P3	3	6	X	18

**Table 2:** Interaction data Moncton Team 2.

**Moncton Team #2**									
**Phase 1**					**Phase 2**				
	P1	P2	P3	Team		P1	P2	P3	Team
P1	X	9	5	21	P1	X	6	3	21
P2	14	X	1	8	P2	7	X	0	17
P3	8	0	X	5	P3	2	0	X	13
**Phase 3**					**Phase 4**				
	P1	P2	P3	Team		P1	P2	P3	Team
P1	X	4	3	26	P1	X	7	5	22
P2	4	X	1	12	P2	15	X	1	20
P3	2	1	X	11	P3	5	1	X	7

**Table 3:** Interaction data Halifax Team 1.

**Halifax Team #1**									
**Phase 1**					**Phase 2**				
	P1	P2	P3	Team		P1	P2	P3	Team
P1	X	4	2	17	P1	X	0	4	16
P2	6	X	2	5	P2	0	X	0	8
P3	5	1	X	3	P3	3	0	X	13
**Phase 3**					**Phase 4**				
	P1	P2	P3	Team		P1	P2	P3	Team
P1	X	3	4	26	P1	X	1	4	14
P2	3	X	2	18	P2	3	X	2	6
P3	4	2	X	20	P3	4	3	X	7

**Table 4:** Interaction data Halifax Team 2.

**Halifax Team #2**											
**Phase 1**						**Phase 2**					
	P1	P2	P3	P4	Team		P1	P2	P3	P4	Team
P1	X	3	2	2	15	P1	X	3	2	3	26
P2	4	X	0	0	8	P2	3	X	1	1	17
P3	4	0	X	0	9	P3	3	1	X	1	17
P4	4	2	0	X	7	P4	3	0	0	X	11
**Phase 3**						**Phase 4**					
	P1	P2	P3	P4	Team		P1	P2	P3	P4	Team
P1	X	3	4	3	23	P1	X	2	2	3	14
P2	3	X	0	0	15	P2	3	X	2	2	6
P3	4	0	X	0	21	P3	3	2	X	3	9
P4	3	0	0	X	18	P4	3	1	3	X	5

**Table 5:** Interaction data Ottawa Team 1.

**Ottawa Team #1**									
**Phase 1**					**Phase 2**				
	P1	P2	P3	Team		P1	P2	P3	Team
P1	X	9	0	9	P1	X	9	3	13
P2	10	X	6	8	P2	8	X	8	14
P3	0	6	X	5	P3	2	5	X	12
**Phase 3**					**Phase 4**				
	1	2	3	Team		1	2	3	Team
P1	X	4	1	12	P1	X	4	4	13
P2	4	X	6	15	P2	7	X	4	17
P3	1	4	X	14	P3	4	5	X	13

**Table 6:** Interaction data Ottawa Team 2.

**Ottawa Team #2**											
**Phase 1**						**Phase 2**					
	P1	P2	P3	P4	Team		P1	P2	P3	P4	Team
P1	X	5	2	0	12	P1	X	1	2	0	10
P2	5	X	3	0	10	P2	1	X	6	0	19
P3	1	3	X	6	18	P3	2	6	X	4	20
P4	0	0	6	X	9	P4	0	1	3	X	14
**Phase 3**						**Phase 4**					
	P1	P2	P3	P4	Team		P1	P2	P3	P4	Team
P1	X	3	2	0	9	P1	X	2	0	0	9
P2	3	X	3	2	18	P2	3	X	3	1	16
P3	0	4	X	6	21	P3	0	3	X	7	19
P4	0	1	6	X	16	P4	0	1	5	X	17

**Table 7:** Interaction data Ottawa Team 3.

**Ottawa Team #3**									
**Phase 1**					**Phase 2**				
	P1	P2	P3	Team		P1	P2	P3	Team
P1	X	6	0	13	P1	X	1	2	22
P2	6	X	3	15	P2	2	X	6	16
P3	0	3	X	8	P3	4	5	X	16
**Phase 3**					**Phase 4**				
	P1	P2	P3	Team		P1	P2	P3	Team
P1	X	2	2	17	P1	X	5	3	19
P2	2	X	5	17	P2	3	X	4	20
P3	2	5	X	14	P3	3	4	X	18

**Table 8:** Interaction data McGill Team 1.

**McGill Team #1**													
**Phase 1**							**Phase 2**						
	P1	P2	P3	P4	P5	Team		P1	P2	P3	P4	P5	Team
P1	X	1	2	1	1	3	P1	X	1	0	0	0	1
P2	1	X	1	2	0	8	P2	1	X	3	1	1	17
P3	1	1	X	5	4	14	P3	0	2	X	4	1	16
P4	0	2	4	X	2	12	P4	0	2	4	X	0	15
P5	1	1	5	2	X	12	P5	0	1	2	0	X	12
**Phase 3**							**Phase 4**						
	P1	P2	P3	P4	P5	Team		P1	P2	P3	P4	P5	Team
P1	X	1	0	0	0	4	P1	X	0	0	0	0	3
P2	1	X	3	0	2	19	P2	0	X	1	2	2	14
P3	0	3	X	1	0	16	P3	0	0	X	1	1	22
P4	0	0	1	X	0	16	P4	0	2	1	X	0	20
P5	0	2	0	0	X	14	P5	1	0	1	0	X	19

**Table 9:** Interaction data McGill Team 2.

**McGill Team #2**											
**Phase 1**						**Phase 2**					
	P1	P2	P3	P4	Team		P1	P2	P3	P4	Team
P1	X	6	3	2	14	P1	X	2	3	1	27
P2	4	X	8	4	13	P2	2	X	5	1	27
P3	3	10	X	3	10	P3	3	5	X	4	25
P4	3	2	4	X	15	P4	1	0	3	X	19
**Phase 3**						**Phase 4**					
	P1	P2	P3	P4	Team		P1	P2	P3	P4	Team
P1	X	10	7	4	16	P1	X	0	5	4	33
P2	7	X	6	4	17	P2	0	X	3	2	30
P3	6	7	X	3	17	P3	4	4	X	5	32
P4	3	2	3	X	16	P4	2	1	3	X	23

**Table 10:** Interaction data McGill Team 3.

**McGill Team #3**									
**Phase 1**					**Phase 2**				
	P1	P2	P3	Team		P1	P2	P3	Team
P1	X	6	2	7	P1	X	2	1	13
P2	6	X	9	14	P2	2	X	7	20
P3	2	10	X	13	P3	1	7	X	22
**Phase 3**					**Phase 4**				
	P1	P2	P3	Team		P1	P2	P3	Team
P1	X	2	2	10	P1	X	5	2	7
P2	2	X	8	23	P2	2	X	11	12
P3	2	7	X	22	P3	2	13	X	11

**Table 11:** Interaction data McGill Team 4.

**McGill Team #4**													
**Phase 1**							**Phase 2**						
	P1	P2	P3	P4	P5	Team		P1	P2	P3	P4	P5	Team
P1	X	1	0	0	0	15	P1	X	1	0	1	0	20
P2	2	X	0	1	1	22	P2	1	X	0	0	0	34
P3	0	0	X	0	4	4	P3	0	0	X	0	0	0
P4	0	1	0	X	2	18	P4	0	0	0	X	1	23
P5	1	1	0	0	X	22	P5	0	0	0	1	X	29
**Phase 3**							**Phase 4**						
	P1	P2	P3	P4	P5	Team		P1	P2	P3	P4	P5	Team
P1	X	0	0	0	0	24	P1	X	2	0	0	0	6
P2	0	X	0	0	0	35	P2	2	X	0	0	0	16
P3	0	0	X	0	0	0	P3	0	0	X	0	0	0
P4	0	0	0	X	0	29	P4	0	0	0	X	4	14
P5	1	0	0	0	X	23	P5	1	0	0	4	X	15

**Table 12:** Team composition.

**Event**	**Participant**	**Teams**	**# Team Members**
**Ottawa (RVCA)**	10	O1	3
		O2	4
		O3	3
**Halifax (SRA)**	7	H1	3
		H2	4
**Moncton (PWA)**	6	M1	3
		M2	3
**McGill**	17	MG1	5
		MG2	4
		MG3	3
		MG$	5

### 2.3 Experimental design and methods

Participants for each event were randomly divided into teams and given the chance to play the Aqua Republica serious game. Participants had no say in which team they were a part of. Participants were recorded while playing the game. The recordings were then analyzed and interactions from all participants were tallied and then transformed into interaction maps divided into four 10-min phases (see data section for information on how phases were divided).
