# Supplementary material for: Interaction analysis data of simulation gaming events using the serious game Aqua Republica
Source: Data Brief. 2018 Jun 27;19:2315–28. doi: 10.1016/j.dib.2018.06.031 (PMC6141151; doi:10.1016/j.dib.2018.06.031)
Supplement: Supplementary file 1 — Supplementary material [file mmc1.docx]

# Paper Title: Interaction analysis data of simulation gaming events using the serious game Aqua Republica

# Manuscript number: DIB-D-18-01205

There are no conflicts of interest to declare.
